# Exploring the Anti-Hypertensive Potential of Lemongrass—A Comprehensive Review

**DOI:** 10.3390/biology11101382

**Published:** 2022-09-22

**Authors:** Henrique Silva, Rita Bárbara

**Affiliations:** 1 Research Institute for Medicines (iMed.ULisboa), Faculdade de Farmácia, Universidade de Lisboa, Av. Prof. Gama Pinto, 1649-003 Lisbon, Portugal; 2 Department of Pharmacy, Pharmacology and Health Technologies, Faculdade de Farmácia, Universidade de Lisboa, Av. Prof. Gama Pinto, 1649-003 Lisbon, Portugal; 3 Biophysics and Biomedical Engineering Institute (IBEB), Faculdade de Ciências, Universidade de Lisboa, Campo Grande, 1749-016 Lisbon, Portugal; 4 Instituto de Saúde Ambiental, Faculdade de Medicina, Universidade de Lisboa, 1649-028 Lisboa, Portugal

**Keywords:** lemongrass, anti-hypertensive, vasorelaxant, cardiovascular, review

## Abstract

**Simple Summary:**

Lemongrass is an herb used in folk medicine for the treatment of hypertension, although its pharmacological potential has not yet been thoroughly studied. This paper provides the first comprehensive review on the anti-hypertensive potential of lemongrass, from in vitro to clinical studies. Even though the composition of lemongrass is dependent on its geographical origin, citral constitutes its major compound. Both citral and lemongrass display vasorelaxant activity ex vivo, promoting the secretion of endothelial vasodilators and the blockage of calcium channels in the vascular smooth muscle. Additionally, citral also displays a negative chronotrope effect. In animal models and in human subjects, lemongrass significantly decreases blood pressure, probably due to the combination of the above-mentioned effects together with diuretic activity. Future preclinical studies are necessary to identify other anti-hypertensive compounds/pathways, as well as to better characterize the safety profile of lemongrass.

**Abstract:**

Lemongrass (*Cymbopogon citratus* (DC) Stapf) is a herb commonly used in folk medicine for many purposes. However, its anti-hypertensive potential has not yet been thoroughly studied. This paper reviews the anti-hypertensive effects of both lemongrass and its main compound citral in in vitro, ex vivo, preclinical and clinical studies. Lemongrass essential oil contains terpenes and their derivatives, whereas extracts contain different classes of polyphenols. Both citral and lemongrass display vasorelaxant activity ex vivo, acting by the promotion of endothelial nitric oxide/prostanoids secretion together with the blockage of calcium channels in the vascular smooth muscle. Citral also displays a negative chronotrope effect, probably due to a centrally mediated enhancement of parasympathetic activity. In both healthy and hypertensive animals, the acute administration of lemongrass results in a decrease in blood pressure, sometimes accompanied by a compensatory increase in heart rate. Similarly, in healthy and hypertensive human subjects, the consumption of lemongrass tea decreases blood pressure. Additionally, a weak/moderate diuretic activity has also been reported in animals and humans, although the mechanisms of action remain elusive. Future preclinical studies are necessary to identify other compounds with anti-hypertensive activity and additional pharmacological pathways. Although well tolerated, the safety profile of lemongrass should be better characterized.

## 1. Introduction

Hypertension is a major cardiovascular disease whose clinical complications are responsible for considerable mortality and morbidity around the world, resulting in increased healthcare costs [1]. Current estimations anticipate an increase in the prevalence of hypertension for the next decade. An important strategy to hinder the progression of hypertension is prevention, either by the promotion of healthy lifestyles, such as regular exercise, adequate nutrition and stress management, or by early diagnosis and pharmacological treatment [2]. Access to medication is a critical aspect of treatment, and is currently not guaranteed for the populations of many low-income countries. It is estimated that more than two thirds of the world’s population is largely dependent on the use of traditional medicines and herbal products for their primary health care needs [3,4]. There are numerous herbal products that have been used for thousands of years as traditional or folk medicines [5]. Interestingly, in higher-income countries, the popularity of so-called ‘alternative therapies’, including phytotherapy, has increased due to the common misconception/perception that ‘natural’ products are safer than conventional drugs [6] or that combining them with conventional drugs will increase treatment efficacy [7].

The genus *Cymbopogon* belongs to the *Poaceae* (*Gramineae*) botanical family and comprises around 180 species, subspecies, varieties, and subvarieties with a wide geographical distribution in tropical and temperate regions around the world [8]. The name *Cymbopogon* is of Greek origin and derives from the words *kymbe* (i.e., boat) and *pogon* (i.e., beard), which relates to the flower spike arrangement of the species of this genus [9]. The genus *Cymbopogon* contains several species, of which *Cymbopogon citratus* (DC) Stapf., commonly known as West Indian or American lemongrass, is the most studied due to its numerous ethnopharmacological applications [9]. In the current paper, this plant will be referred to simply as lemongrass.

**Figure 1:**
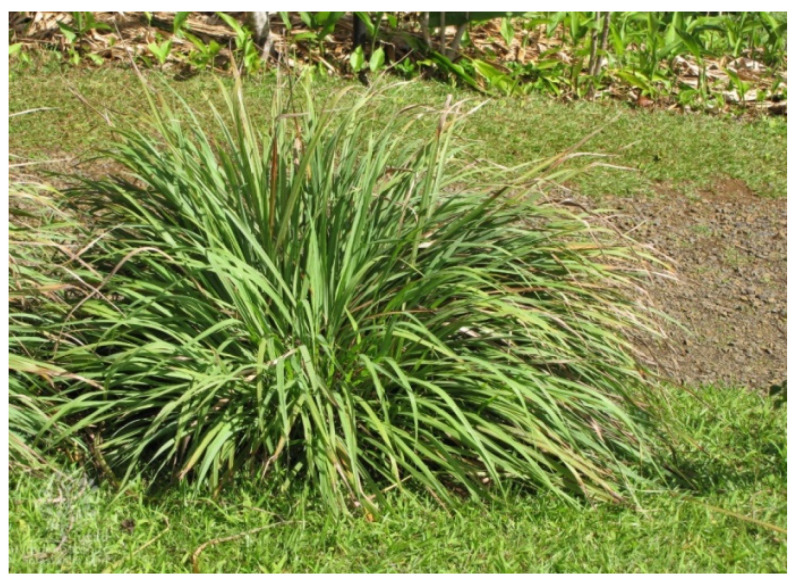
Aerial parts of *Cymbopogon citratus* (DC) Staph. Photograph by Forest and Kim Starr from Jardim Botânico UTAD, Flora Digital de Portugal (https://jb.utad.pt, accessed on 1 August 2022).

Lemongrass is a tall perennial and fast-growing herb displaying a tuft of leaves that sprout from annulate and sparingly branched rhizomes (Figure 1). It has many bulbous stems that increase the bulk size of the plant as it grows [9], and can reach a height greater than 2 m and a width of around 1 m. [10]. The leaves are distinctively bluish-green, lemon scented (justifying the name of the herb) [11], with a width of 5–15 mm, and do not produce seeds. Lemongrass likely originates from India or Sri Lanka and it is nowadays cultivated in tropical and subtropical humid regions of North and South America, Europe, North Africa, the Indian subcontinent and Australia [12]. It is known by many different common names wherever it is used, as reviewed elsewhere [12]. The appealing citrus aroma of the plant has justified its use in the cosmetic industry and in the development of perfumes. In Latin American, African and Asian countries, lemongrass leaves and stems have been used as a culinary flavoring agent in curries, soups, seafood and poultry [13,14,15]. The leaves are extensively used in herbal teas as a lemon-flavoring ingredient, as well as for other popular drinks [14,16]. In the food industry, it has recently been explored as a food preservative [17]. Lemongrass is also an abundant source of lignocellulose, a raw material used for the production of paper and pulp [18], silica [19], composites and adsorbing materials [20,21], and bioenergy [22]. For medicinal purposes, the most relevant parts of the plant are the leaves, which can be used fresh or dried, although the stems and rhizomes have also been explored [12]. From these parts, different products can be made, namely, water or alcohol extracts, as well as essential oils.

Lemongrass has been employed as a folk medicine in many countries for a variety of purposes. In fact, several biological activities have been reported throughout the years from scientific studies, including antibacterial [9], antifungal [23], antiprotozoal [24], anti-inflammatory [25], antioxidant [26] and anti-carcinogenic activities [26], among others. Given its vast array of applications, it is not surprising that the popularity of lemongrass has increased in recent years, with an increasing number of scientific publications in the last two decades [27]. Several of these publications mention that lemongrass has been used as a folk medicine to lower blood pressure in different countries such as Spain (Canary Islands) [28], Cuba [29,30], Cameroon [31], Egypt [32] and Brazil [33,34].

Several reviews on the biological activities of lemongrass have been published in recent years [10,13,35,36,37,38,39,40,41,42]; however, the cardiovascular potential of this plant is covered only broadly. To the authors’ knowledge, the current paper constitutes the first comprehensive review on the anti-hypertensive potential of lemongrass. By providing a thorough description of the different pharmacological mechanisms underlying this anti-hypertensive activity, this review aims to establish a rationale for drug design as well as to improve the conception of future pre-clinical and clinical studies. Even though other *Cymbopogon* species also display significant beneficial cardiovascular activity, the review of their potential is beyond the scope of this paper. Similarly, the cardiovascular activities of minor lemongrass compounds are also not analyzed.

## 2. Chemical Characterization of Lemongrass and Its Major Bioactive Compounds

### 2.1. Composition of Lemongrass Products

Two main products with biological activity can be obtained from lemongrass: extracts and essential oils, which differ substantially in composition. These products have been explored for their cardiovascular potential in vitro, ex vivo and in vivo.

Lemongrass essential oil corresponds to 1–2% of the total dry weight of the herb [43] and consists mainly of terpenes and terpenoids (alcohols, ketones and esters). An extensive list of the compounds identified in lemongrass so far can be consulted in recent reviews [10,44]. The composition of essential oils from different geographical origins are displayed in Table 1. In addition, minerals such as potassium, calcium, silica and phosphorus are also present [19], as well as vitamins A, B_2_ (riboflavin), B_3_ (niacin), B_6_ (pyridoxine), B_9_ (folate), and E and protein, carbohydrates and fat [45].

**Figure 2:**
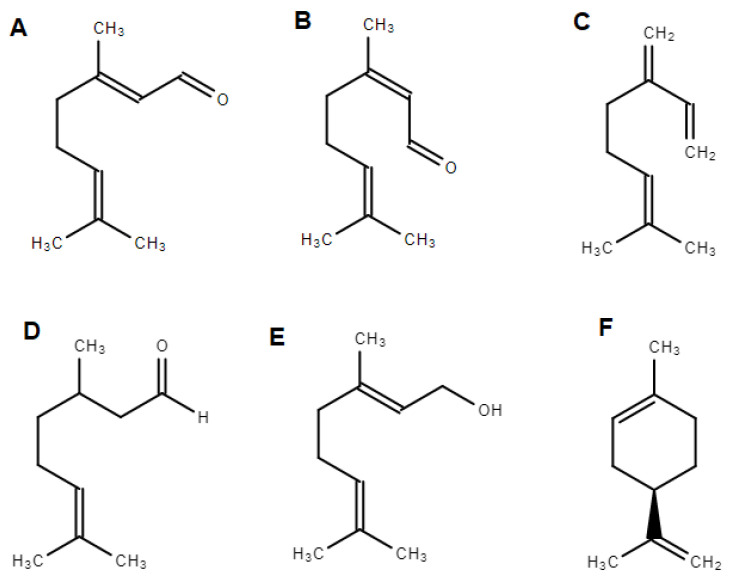
Chemical structures of the most prevalent bioactive compounds identified in lemongrass essential oil: geranial (**A**) and neral (**B**), whose mixture constitutes citral, β-myrcene (**C**), citronellal (**D**), geraniol (**E**) and limonene (**F**). These structures were drawn by Chem Spider Draw software.

The two major compounds that have been identified from the essential oil of lemongrass are citral and β-myrcene. Citral is a mixture of two stereoisomer monoterpene aldehydes: geranial (i.e., *trans*-citral, E-citral, α-citral, citral A or E-3,7-dimethyl-2,6-octadienal), which generally predominates, and neral (i.e., *cis*-citral, Z-citral, β-citral, citral B or Z-3,7-climethyl-2,6-octadienal) [44]. When citral is synthesized in amounts that reach 75% of the plants’ content, it produces a high-quality oil [52]. Samples from Brazil, Africa and Asia usually display high contents of citral [44], although Ethiopian [53] samples have exceptionally shown the opposite. β-Myrcene content is strongly related to the geographical origin of lemongrass, and can vary from 0.8% in samples from Egypt [54] to 25% in samples from Brazil [55] and Nigeria [49]. In addition geraniol, citronellal, and limonene are also often identified in levels above 1% in some samples, even though they cannot be considered marker compounds, since their levels also vary considerably [44]. For example, Nigerian samples are found to be rich in limonene (7.90%) [56], whereas the Kenyan samples lack this terpene [57]. Citronellal can range from 0.12% in Kenyan samples [57] to 12.77% in Nigerian samples [56]. Geraniol is the most prevalent alcohol and can range from 1.34% in South African samples [58] to 21.86% in Nigerian samples [56] to 40% in Ethiopian samples [59]. Interestingly, one study has identified two triterpenoids, cymbopogone and cympopogonol [60,61], which have not been described elsewhere since the time of the original publication. The chemical structures of the most prevalent bioactive compounds in lemongrass essential oil are represented in Figure 2.

The composition of lemongrass essential oil is affected by several factors, including geographical location [44], soil composition [47,62], farming practices [44], plant age [63], proportion of young leaves to older leaves during harvesting (i.e., determines the high citral content) [64], photoperiod [44], as well as harvesting (manual vs. mechanical) [47,65], drying (air-dried vs. oven-dried) [66] and extraction methods [44]. The drying method strongly influences the essential oil composition. In fact, one study has shown that the essential oil yield was higher in oven-drying at 45 °C than drying in the sun or in the shade [66]. The age at which the plant is harvested also affects its composition. One study reported a great variability in the number and concentration of compounds present in the essential oil at different plant ages, having identified 44 compounds at 4.5 months and 50 at 7.5 months, but only 15 at 6.5 months [64]. Different drying methods can affect the percentage of citral by more than 8% [66].

Typically, lemongrass extracts are made using water (infusions and decoctions), methanol or ethanol as solvents. In contrast to essential oils, lemongrass extracts contain nonvolatile compounds such as alkaloids, saponins, tannins, anthraquinones, steroids, proanthocyanidins, phenolic acids and flavonoids, although few studies identify them specifically [67]. The main phenolic acids described have been hydroxycinnamic acids (derivatives of caffeic and p-coumaric acids, together with chlorogenic, ferulic and rosmarinic acids) and hydroxybenzoic acids (gallic acid). The main flavonoids described are glycosides of luteoline and apigenin (flavones), glycosides of quercetin and isoquercetin (flavonols), as well as catechin (a flavan-3-ol), eriodictyol (a flavanone) and swertiajaponin (a C-glycoside flavonoid) [68]. Of the tannins mentioned, only tannic acid has been described in the literature [68]. The main polyphenols identified for the different types of extracts are described in Table 2.

### 2.2. Chemical Characterization and Metabolism of the Lemongrass and Its Main Compounds

Citral is a clear yellow liquid that is highly volatile and poorly soluble in water. It has a molecular weight of 152.23 g/mol and a log P (octanol/water) of ~3 [74]. In rodents, citral is rapidly absorbed and metabolized by the liver following oral or dermal exposure [75], and is predominantly excreted in the urine [76]. In addition, it is thought that geranial is metabolized faster than neral [77]. In Sprague-Dawley rats, citral does not produce developmental toxicity when administered by inhalation (6 h/day during gestation days 6–15) at concentrations below 68 ppm, which was determined to be the maternally toxic exposure level [78]. In normal rat liver epithelial cell lines (RL34 cells), it was found that citral could induce the activity of glutathione S-transferase [79]. Similar results were found in a recent animal study. When administered orally to Sprague-Dawley rats at a dose of 240 mg/kg for two weeks, citral decreased the activity of phase I liver enzymes and increased the activity of phase II enzymes, although it did not markedly affect the concentration of alanine aminotransferase induced by acetaminophen [80]. These results suggest that citral can modulate drug-metabolizing enzymes and thereby affect the pharmacological activity of drugs and their toxicity profile.

Few data exist on the effect of lemongrass products on metabolism. In Sprague-Dawley rats receiving lemongrass oil at 200 mg/kg or 400 mg/kg for two weeks, the activity of phase I liver enzymes decreased and the activity of phase II enzymes increased, although the concentration of alanine aminotransferase induced by acetaminophen remained unaltered [80].

## 3. Cardiovascular Activity of Lemongrass and Citral

### 3.1. Antioxidant and Anti-Inflammatory Activities of Citral and Lemongrass In Vitro

Of the few studies published so far on the effects of citral and lemongrass on cell cultures, antioxidant and anti-inflammatory activities are the most reported. In human umbilical vein endothelial cells (HUVECs), preincubation with citral significantly inhibits the expression of vascular cell adhesion molecule (VCAM)-1, intercellular adhesion molecule (ICAM)-1, tumor necrosis factor alpha (TNF-α), interleukin (IL)-8 and nuclear factor kappa B (NF-κB) after exposition to lipopolysaccharide (LPS). This effect is attributed to the activation of peroxisome proliferator-activated receptor (PPAR)-γ [81]. Similarly, in macrophages from BALB/c mice, citral inhibits the release of IL-6, IL-1β and IL-10 [82], and in human macrophage-like U937 cells, it suppresses LPS-induced cyclooxygenase-2 mRNA and protein expression while increasing the mRNA expression of PPARα and PPARγ-sensitive genes [83]. Therefore, the activation of PPAR proteins seems to play an important role on the anti-inflammatory activity of citral. Additionally, citral also displays antioxidant activity, having been shown to protect HUVECs against hydrogen peroxide-mediated oxidative stress [84].

Lemongrass extracts and essential oils also display significant anti-inflammatory activity. In HUVECs, a lemongrass extract inhibited the production of reactive oxygen species (ROS) evoked by pretreatment with hydrogen peroxide, high glucose, and oxidized low-density lipoprotein (oxLDL) [72]. In BALB/c mice macrophages exposed to LPS, lemongrass essential oil decreases the secretion of IL-1β and IL-6 [85]. In LPS-exposed raw 264.7 macrophages, a lemongrass extract inhibited the inducible nitric oxide synthase (iNOS) expression, NO production, as well as several pro-inflammatory pathways (MAPK, JNK and NF-κB) [25]. Finally, fractions of lemongrass extracts also display notable activity. A polysaccharide-rich fraction of lemongrass extract inhibited NO and ROS production in LPS-exposed raw 264.7 macrophages [86]. In BALB/c mice macrophages, a lemongrass extract decreased the LPS-mediated secretion of IL-6 but not of IL-10 [82].

### 3.2. Vasorelaxant Activity of Citral Ex Vivo

**Figure 3:**
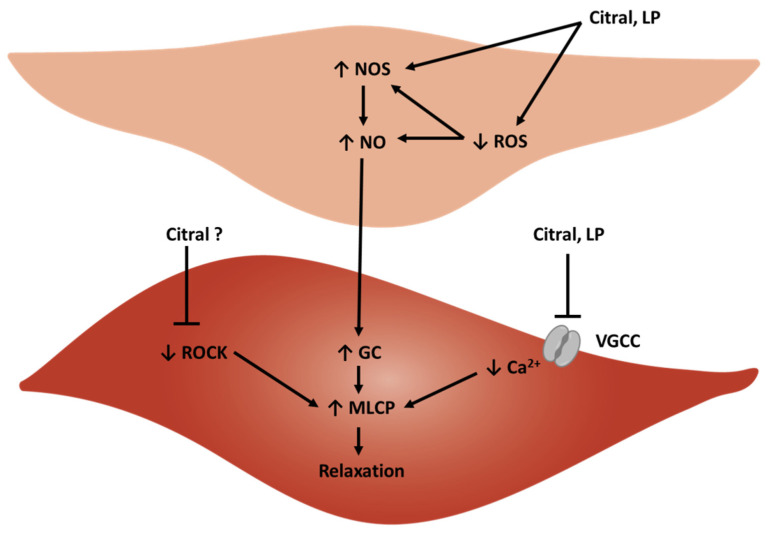
Scheme of the probable vasorelaxation mechanisms of citral and of lemongrass products (LP). An endothelial cell is represented on top and a vascular smooth muscle cell at the bottom (GC—guanylyl cyclase; MLCP—myosin light-chain phosphatase; NO—nitric oxide; NOS—nitric oxide synthase; ROCK—Rho-associated protein kinase; ROS—reactive oxygen species; VGCC—voltage-gated calcium channel).

The vasorelaxant activity of citral has been investigated in the aorta and superior mesenteric arteries of healthy and hypertensive rats [87,88,89] and the main results are presented in Table 3. The probable vasorelaxant mechanisms of citral are represented in Figure 3. Three studies have evaluated the potential vasorelaxant activity of citral in the aorta of healthy Wistar–Kyoto rats, with the studies of Pereira et al. (2013) and Moreira et al. (2013) showing significant activity [88,89]. Citral evoked a dose-dependent vasorelaxation of phenylephrine (PE)-precontracted aortae. Comparable magnitudes were detected in both endothelium-intact and endothelium-denuded vessels, suggesting that citral evokes vasorelaxation independently of the endothelium. In addition, citral also inhibits arterial contraction evoked by calcium chloride (CaCl_2_) and by potassium chloride (KCl), which depolarize vascular smooth muscle (VSM) cells. Taken together, these experiments have clarified that citral induces vasorelaxation in the rat aorta by blocking voltage-gated calcium channels (VGCC) in the VSM [88]. In contrast, in the study performed by Devi et al. (2012), no vasorelaxant activity was detected [87]. There are some differences in terms of experimental design between two of these studies, which might affect the results. On the one hand, the concentration of PE for aortic precontraction was different in these studies (1 μM in Devi et al. vs. 10 μM in Pereira et al.). On the other hand, the age of the animals was probably different, with Pereira et al. using 15–17-week-old animals, compared to Devi et al. who likely used slightly younger animals judging from their weight (i.e., 250–300 g corresponds to 10–15 w.o.) [90]. The concentration of citral used was comparable between studies and, thus, should not be responsible for this discrepancy. Finally, it should also be considered that since citral is a mixture of geranial and neral, it is possible that these stereoisomers display different vascular activities, as demonstrated for other isomeric compounds [91].

Moreira et al. (2013) also explored the vasorelaxant activity of citral in the superior mesenteric artery of Wistar rats [89]. Endothelium denudation and incubation with tetraethylammonium (TEA, i.e., nonselective potassium channel blocker) did not affect the vasorelaxation of PE-precontracted vessels, showing that citral acts on VSM. There was no difference in vasorelaxation between endothelium-denuded vessels and endothelium-intact vessels contracted with KCl. In addition, citral also abolished contraction induced by CaCl_2_ and by sodium orthovanadate (i.e., inhibitor of protein tyrosine phosphatase), meaning that citral acts in the VSM by blocking L-type VGCC and/or diminishing the calcium sensitivity of contractile proteins. It is currently known that sodium orthovanadate inhibits myosin light-chain phosphatase by activating Rho-kinase (ROCK) [92], and it is possible that citral inhibits the latter enzyme.

In spontaneously hypertensive rats, citral inhibits PE-mediated aortic contraction, an activity which is partly attenuated by L-N^ω^-nitro arginine methyl ester (L-NAME, i.e., a nitric oxide synthase inhibitor). This shows that citral acts in part by increasing the endothelial secretion of NO in this particular strain. However, preincubating the aorta with indomethacin (i.e., a non-specific cyclooxygenase inhibitor) does not affect the vasorelaxant activity of citral, showing that this compound does not act by inducing the endothelial secretion of prostanoids. Similarly to PE-precontracted vessels, citral also attenuates CaCl_2_-induced aortic contraction, showing that it also acts on VSM cells, possibly by blocking receptor-mediated or VGCC on the plasma membrane and/or sarcoplasmic reticulum [87].

Several other lemongrass bioactive compounds such as citronellol [93,94], citronellal [95] and linalool [96,97] also display vasorelaxant activities. Considering their structural similarity to citral, it is safe to assume they share a similar chemical moiety that is largely responsible for their effects, synergic and complementary, which could serve as a basis for the development of new drugs.

### 3.3. Anti-Hypertensive Activity of Citral In Vivo

In healthy conscious Wistar rats, the intravenous administration of citral (1–20 mg/kg) creates transient hypotension accompanied by bradycardia [77]. Whereas the hypotensive response was only reduced by administration of atropine, bradycardia was abolished, meaning that citral acts by cardiac depression and by peripheral vasodilation. At 10 and 20 mg/kg, citral reduced the QTc interval in electrocardiographic recordings, suggesting a potential effect on myocardial potassium channels, and at 20 mg/kg it increased QRS duration, showing an arrhythmogenic potential. The administration of L-NAME, but not of indomethacin, attenuated the hypotensive response, but not bradycardia, confirming that peripheral vasodilation is endothelial NO-dependent. The administration of hexamethonium (i.e., ganglionic nicotinic receptor antagonist) attenuated both hypotensive and bradycardic responses, suggesting that citral induces cardiac depression either via the activation of muscarinic receptors or by vagal activation. Finally, the administration of citral to animals anesthetized with sodium thiopental (i.e., barbiturate central depressor with vagolytic activity) attenuated hypotension and bradycardia, further suggesting that citral acts centrally. Curiously, citronellol has been found to induce vasodilation in rats in part due to a vagovagal reflex, which further supports the same mechanism of action for citral [86].

### 3.4. Cardiac Activity of Lemongrass Products Ex Vivo

The application of a lemongrass water extract to isolated rat hearts provokes a rapid and significant decrease in heart rate, but not in contractile force. The authors attributed this response to the stimulation of myocardial muscarinic receptors and/or to the blockage of VGCC, which could be evoked by the alkaloids of the extract [71]. As shown above, it is possible that the blockage of VGCC may be evoked by citral, although other bioactive compounds may show this activity as well. Although some studies have reported the existence of alkaloids in lemongrass, they have failed to specify which ones. Therefore, a more thorough chemical characterization of lemongrass extracts is needed in order to explain its cardiac effects.

### 3.5. Vasorelaxant Activity of Lemongrass Products Ex Vivo

The vasorelaxant activity of lemongrass extracts and essential oils have been explored in both animal [87,98,99,100] and human arteries [73], as well as in human veins [72], with considerable variability between them. The main results of these studies are summarized in Table 4. The probable vasorelaxation mechanisms of lemongrass products are represented in Figure 3.

Methanolic extracts of lemongrass induced vasorelaxation of PE-precontracted [87] and of norepinephrine (NE)-precontracted aorta of Wistar-Kyoto rats [98,99]. In the study by Devi et al., no specific vasorelaxation mechanisms were characterized [87]. In the study by Abeywardena et al. this response was significantly decreased by incubation with N^ω^-nitro-L-arginine (NOLA, i.e., nitric oxide synthase inhibitor) and by endothelium denudation [99], whereas in the study by Runnie et al. only NOLA was able to decrease lemongrass-induced vasorelaxation [98]. In Wistar rats, a hydroalcoholic tincture of lemongrass induced vasorelaxation of endothelium-denuded aortae precontracted with PE or KCl. Taken together, these results suggest that lemongrass induces vasorelaxation by potentiation of endothelial-dependent NO secretion as well as by an endothelium-independent mechanism, probably by acting on VSM calcium channels.

In the PE-precontracted superior mesenteric artery of Wistar-Kyoto rats methanolic extracts of lemongrass exhibited a higher vasorelaxation activity than in the aorta [98,99]. The study by Abeywardena et al. showed that vasorelaxation was partially decreased by the incubation with NOLA and indomethacin, although not significantly [99]. In contrast, the study by Runnie et al. showed a significant decrease by L-NAME and indomethacin [98]. Considering that in both studies there was a partial decrease in the vasorelaxant activity of lemongrass, it is only logical to assume that there are other vasorelaxation mechanisms involved, endothelial NO/prostanoid-independent and/or mediated by modulation of VSM calcium-dependent pathways.

In human internal thoracic arteries, a water extract of lemongrass, found to contain flavonoids, phenolic acids and tannins, caused different effects depending on the concentration [73]. Preincubation with 0.002 mg/mL and 2 mg/mL extracts caused a potentiation of NE-mediated contraction, whereas with 0.0002 mg/mL, the contractility was found to decrease. Preincubating the arteries with 0.2 and 1 mg/mL phenolic acid fraction or with 1 mg/mL tannin fraction potentiated NE-induced contraction, whereas preincubation with 0.2 mg/mL tannin fraction or with 0.2 and 1 mg/mL flavonoid fraction decreased that contraction. This suggests that phenolic acid and/or tannin fractions contain vasoconstrictive bioactive compounds, as suggested in a previous study [75]. In addition, in NE-precontracted arteries, the phenolic acid fraction increased that contraction, whereas the tannin fraction decreased it, revealing vasorelaxant activity. The addition of indomethacin abolished the vasorelaxant activity of the extract, but not that of the tannin fraction. Taken together, these results suggest (1) the existence of both vasoconstrictor and vasorelaxant compounds in the leaves of lemongrass, (2) that the dominant vascular effect seems to be strongly dependent on the concentration, and (3) the vasorelaxant activity of tannins is not dependent on the endothelial secretion of prostanoids.

Finally, in human umbilical veins precontracted with U46619 (i.e., thromboxane A_2_ receptor agonist), an antioxidant-rich fraction of a hydromethanolic extract of lemongrass caused relaxation, suggesting an endothelium-independent activity, probably via the modulation of calcium intracellular stores and/or its influx from the extracellular medium [72].

In conclusion, these studies show that depending on its composition, lemongrass extracts cause vasorelaxation via mechanisms dependent and independent of the endothelium, possibly via the modulation of intracellular calcium concentration.

### 3.6. Anti-Hypertensive Activities of Lemongrass In Vivo

#### 3.6.1. Vasodilation and Cardiac Suppression Activity

The beneficial anti-hypertensive activity of lemongrass extracts and essential oils have been observed with both oral and intravenous administration to conscious and anesthetized animals. The main results of these studies are summarized in Table 5.

When administered intravenously, a single dose of water extract of lemongrass causes an immediate and significant reduction in blood pressure in anesthetized Wistar rats. For 1 and 2 mL/kg doses, this effect was transient, lasting approximately 6 and 15 min, respectively. Alternatively, the 3 mL/kg dose lasted for more than 35 min [29]. In a similar study, the intravenous administration of a hydroalcoholic lemongrass extract at 5 mg/mL (1 mg in 0.2 mL), corresponding to a 0.8 mg/kg dose, caused a fast and short-lived (less than 2 min) decrease in blood pressure [73]. The lower duration of effect in this study is probably related to the much lower dose used in this study when compared to that by Carbajal et al. (1989), although a rapid metabolism of the bioactive compounds must also be considered. Considering that heart rate was not measured in these studies, it is unclear whether this hypotensive effect was caused by vasodilation, cardiac depression, or both. However, in a different study, the intravenous administration of a hydroalcoholic extract of lemongrass to anesthetized dogs caused a non-significant increase in blood pressure together with a significant decrease in heart rate from 1.5 to 2.5 h after administration. In this experimental setting, it is unlikely that the cardiac effect is attributed to the negative chronotropic activity of the extract, but rather attributed to the activation of a baroreflex [101].

Several studies have also studied the effects of combinations of lemongrass extract with other plant extracts [31,102]. The intravenous administration of a combination of lemongrass and garlic extracts to anesthetized Wistar rats caused an acute decrease in mean blood pressure, even though the effect was short-lived (<1 min) [102]. In a different study, a water extract of lemongrass, avocado, citrus and honey was orally administered to ethanol and sucrose-induced hypertensive anesthetized Wistar rats. This mixture caused a significant decrease in systolic, diastolic and mean blood pressure, together with a significant decrease in heart rate. Finally, in ethanol and salt-induced hypertensive Wistar rats, the oral administration of 200 mg/kg water extract of lemongrass or of a mixture of lemongrass and hairy beggarticks (*Bidens pilosa* L.) for 7 weeks significantly decreased systolic, diastolic and mean blood pressure [103]. Even though these effects cannot be solely attributed to the bioactive compounds of lemongrass per se, they suggest that these herbal combinations show efficacy to decrease blood pressure, paving the way for the development of new combinations to be explored in the future [31].

The essential oil of lemongrass displays similar activity to that of the extract. The intravenous administration of a single dose of lemongrass essential oil to conscious Wistar rats resulted in a significant decrease in blood pressure and heart rate. The observed bradycardia was less pronounced when the animals were pre-treated with atropine (i.e., muscarinic receptor antagonist). Additionally, bradycardia was less pronounced in animals anesthetized with sodium thiopental, a drug known to have vagolytic and central nervous system depressant activity. Therefore, it appears that certain bioactive compounds in the lemongrass essential oil may act as cardiac muscarinic agonists, as suggested by ex vivo studies [71], in addition to inducing some level of central depression which consequently decreases heart rate. The hypotensive effect, however, was practically unaffected by anesthesia or muscarine, showing that the essential oil of lemongrass also evokes vasodilation with a decrease in peripheral vascular resistance. Since the hypotensive response was not different, regardless of whether the essential oil was administered alone or with L-NAME or indomethacin, it seems that the endothelial release of NO or prostanoids did not contribute to vasodilation [104].

**Table 5:** Main results of in vivo animal studies describing the cardiovascular activities of lemongrass products (i.p.—intraperitoneal; i.v.—intravenous; LDL—low-density lipoprotein; w.o.—weeks old).

Authors	Animal Species/Strain	Lemongrass Product (Dose/Administration)	Main Results
Somparn et al. (2018) [68]	Healthy male Sprague-Dawley rats (N = 30, undisclosed age and weight)	Water extract of the whole plant (250, 500 and 1000 mg/kg/bw/day *per* os for 30 days)	Significant decrease in total cholesterol, LDL and atherogenic index. Significant increase in serum antioxidant activity and decrease in lipid peroxidation.
Arome et al. (2014) [105]	Healthy Swiss albino mice (N = 5, 18–30 g, both sexes, undisclosed age)	Water extract of roots (200, 400 and 600 mg/kg)	Significant reduction in anxiety behavior (reduced body temperature in stress-induced hyperemia model; increased time spent in the open arm in an elevated plus maze model; increased locomotion and decreased rearing and defecation in an open-field model).
Carbajal et al. (1989) [29]	Healthy Wistar rats (N = 5, 180–220 g, undisclosed age) under sodium pentobarbital anesthesia	Water extract of leaves (1, 2 and 3 mL/kg i.v.)	Significant decrease in blood pressure.
Singi et al. (2005) [102]	Healthy Wistar rats (N = 7, 400 g, undisclosed age) under sodium pentobarbital anesthesia	Water/ethanol extract of fresh leaves (aprox. 0.8 mg/kg)	Significant and short-lived decrease in mean blood pressure.
		Water/ethanol extract of fresh lemongrass leaves and garlic bulbs (aprox. 0.8 mg/kg of each)	Significant and short-lived decrease in mean blood pressure
Moreira et al. (2010) [104]	Healthy male Wistar rats (200–300 g, undisclosed number and age)	Essential oil of fresh leaves (5–20 mg/kg i.v.)	Bradycardia fully opposed by atropine and partially by sodium thiopental, but not by L-NAME or indomethacin. Hypotension fully opposed by atropine but not by L-NAME or indomethacin.
Dzeufiet et al. (2014) [31]	Healthy (N = 6) and ethanol/sucrose-induced hypertensive (N = 6) Wistar rats (6–8 w.o., 150–160 g) under urethane anesthesia	Water extract of fresh leaf of avocado, fresh leaves and stems of lemongrass, citron and honey (50, 100 and 150 mg/kg, respectively)	Significant reduction in heart rate, systolic, diastolic and mean blood pressure in comparison with ethanol/sucrose-induced hypertensive animals.
Jutabha et al. (1995) [101]	Mongrel dogs (N = 5, 12–18 kg) anesthetized with sodium pentobarbital (25 mg/kg i.v.)	Leaves (1.25, 2.5, 5.0 and 10 g/kg administered orally)	Significant decrease in heart rate from 1.5 to 2.5 h after administration, probably due to baroreflex. Non-significant increase in blood pressure.
Tcheutchoua et al. (2022) [103]	Male Wistar rats (6–8 w.o., 150–160 g, undisclosed number)	Water extract of leaves and stems (200 mg/kg administered orally (1/day) for 7 weeks)	Significant decrease in systolic, diastolic and mean blood pressure.

#### 3.6.2. Central Nervous System-Depressing Activity

It has been theorized that lemongrass products may decrease blood pressure by their central nervous system-depressant activity, which contributes to decreasing the stress response. A recent study subjected Swiss albino mice to three anxiety-inducing protocols after the administration of 200, 400 and 600 mg/kg of a water extract of lemongrass. In most instances, extracts were found to significantly reduce behavioral anxiety manifestations similarly to diazepam [105]. Even though no cardiovascular variables were measured in this study, it is reasonable to assume that such a decrease in the anxiety response would have been associated with a decrease in blood pressure, possibly due to the suppression of stress-mediated sympathetic effects on the heart and/or vasculature. More studies are necessary to explore this hypothesis.

#### 3.6.3. Diuretic Activity

Several studies have shown that lemongrass products exhibit a diuretic effect, which may also contribute to a decrease in blood pressure [29,103]. In one study, a single dose of a water extract of lemongrass was administered orally to Wistar rats and a weak diuretic activity was observed, even though no cardiovascular variables were measured and only a urinary index was calculated [29]. In ethanol and salt-induced hypertensive Wistar rats, the oral administration of a 200 mg/kg water extract of lemongrass or the mixture of lemongrass and hairy beggarticks extract (200 mg/kg) for 7 weeks showed slightly higher diuretic activity than furosemide (10 mg/kg), significantly increasing urinary volume after 24 h administration [103].

### 3.7. Clinical Studies—Cardiovascular Activities in Humans

Several clinical studies have reported significant anti-hypertensive activity from lemongrass tea (water extract) and essential oil in healthy and hypertensive subjects. The main results of these studies are summarized in Table 6. In one study, young subjects with high anxiety levels were submitted to the Stroop Color and Word test, 30 min after having consumed 150 mL of lemongrass tea. The results showed no difference in terms of pulse rate between these subjects and non-anxious control subjects [33]. Another study was conducted where a group of young, healthy Black subjects drank lemongrass tea (2, 4 or 8 g in 150 mL/day) for 30 days [69,106]. Overall, a decrease in systolic, diastolic and mean blood pressure was observed together with an increase in heart rate. Diastolic blood pressure was the variable that changed the most, with all doses leading to a decrease on day 10, supporting preclinical results that lemongrass products decrease peripheral vascular resistance. In contrast, systolic pressure remained unchanged on day 10. This uneven decrease in blood pressure caused an increase in pulse pressure on day 10 and was accompanied by an increase in heart rate. On day 30, however, only the systolic pressure remained lower than baseline, which was only found with the highest dose; all other parameters had returned to baseline. These authors also assessed the effect of tea consumption on renal function and plasma pH. Diuresis was found to be increased on day 10 with all doses, whereas natriuresis only increased with the highest dose. On day 30, only the 8 g dose was able to maintain diuresis higher than baseline, with the same being observed in natriuresis for the 2 and 8 g doses [69]. In addition, groups treated with either 4 or 8 g of lemongrass extract showed a significant increase in plasma and urine creatinine levels, together with an increase in plasma urea and a decrease in estimated glomerular filtration rate. Finally, in the 8 g treatment group, plasma pH decreased significantly to values compatible with acidemia [106]. These results suggest that these blood-pressure-lowering effects may not be long-lasting, even though they may be negatively affected by a pH-induced change in vascular reactivity [93], as well as by a possible certain inter-group heterogeneity in the study design.

The effects of lemongrass tea in hypertensive subjects have been scarcely studied. In the study by Sobha (2014), a group of pre-hypertensive and hypertensive subjects drank 250 mL of a lemongrass decoction once daily for 14 days [107]. The results showed a significant decrease in systolic blood pressure. However, diastolic blood pressure, heart rate, or other cardiovascular variables were not acquired, which implies that these data should be interpreted with caution.

Because lemongrass oil is perceived as pleasant [46,108] and displays a relaxing activity, it has been employed in aromatherapy in different therapeutic contexts. In one study, a group of young, healthy subjects was asked to inhale the aroma of lemongrass oil, after which they were subjected to the Stroop Color and Word test to evoke mental stress. The results showed that subjects who inhaled lemongrass oil showed a reduced post-Stroop anxiety response after inhalation in comparison to groups who inhaled a different herbal aroma or no aroma at all. However, no significant changes in heart rate were noted between groups [46]. These results are in line with the ones of another study in which cognitive and cardiovascular parameters were assessed in young, healthy female subjects before and after the inhalation of lemongrass essential oil for 5 min. Although subjects showed higher calmness and alertness scores when compared to a control group, no changes in blood pressure or heart rate were detected [109]. Recently, a patent for an aspersion system that creates an atmosphere of citral and linalool (10:1 ratio) to be inhaled with the intent of lowering blood pressure has been created [110].

The relaxing effects of lemongrass oil have also been explored in the context of therapeutic massage [51,108,111]. In one study, a group of subjects with ages ranging from 18 to 82 years old received a therapeutic massage using lemongrass as a vehicle. Massage with lemongrass oil produced a decrease in diastolic blood pressure, which was comparable to other massage vehicles, but caused no relevant changes in systolic blood pressure or in heart rate. Since this study did not compare the hypotensive effect between different massage groups, it is not possible to suggest an effect of the lemongrass oil, especially when it is much more likely that it was the massage itself that caused this effect. Therefore, these results merely demonstrate the interest and suitability in using lemongrass oil as a therapeutic massage vehicle [51]. Citral, the major component of lemongrass oil, is known to undergo transdermal absorption. However, it is presently unclear whether that or other compounds could suffer transdermal absorption when present as part of essential oils, especially considering that they strengthen the structure of the epidermis [112]. Therefore, at present, it is difficult to entertain the hypothesis that lemongrass oil evokes a relaxing effect due to the transdermal absorption of its components. In a different study, a mixture of lemongrass, patchouli and ylang-ylang essential oils was used in a group of young and stress-prone female subjects during an effleurage therapeutic massage. In addition to decreasing mean blood pressure and heart rate, this mixture also further decreased the subjects’ anxiety response to a stress-inducing test compared to other massage modalities [111].

**Table 6:** Main cardiovascular effects of lemongrass products in human subjects.

Authors	Study Sample	Lemongrass Product	Administration Route	Main Cardiovascular Effects
Leite et al. (1986) [33]	Young and healthy subjects (N = 9, 18–35 y.o., undisclosed sex ratio)	Tea made from infusion of and powdered leaves	Oral, once	No difference in pulse rate after subjected to the Stroop test in comparison with controls.
Ekpenyong et al. (2016) [69]	Young healthy subjects (N = 105, both sexes)	Tea made from infusion of powdered leaves	Oral, once daily for 30 days	Mean and diastolic blood pressure, pulse pressure and heart rate decreased on day 10 and day.
Sobha (2014) [107]	Pre-hypertensive and hypertensive subjects (N = 60, both sexes)	Tea made from leaves decoction	Oral, 250 mL once a day for 14 days	Significant decrease in systolic blood pressure.
Goes et al. (2015) [46]	Young healthy subjects (N = 40 males, 18–30 y.o.)	Essential oil	Inhalation (3 deep breaths) of 3 or 6 drops in a paper	Significant decrease in anxiety levels. No significant change in heart rate.
Kamkaen et al. (2015) [51]	Healthy subjects (N = 8 males and 21 females, 18–82 y.o., mean age 50.48 y.o.)	Essential oil	Inhalation of essential oil applied to the skin during massage	Significant decrease in diastolic blood pressure in all groups. Comparison of effect was not performed against control groups.
Siahaan et al. (2014) [111]	Young and healthy but stress-prone female subjects (N = 20, undisclosed age)	3% suspension of ylang-ylang, lemongrass, and patchouli essential oil mixture	Inhalation during massage therapy	Significant decrease in mean blood pressure, larger than control subjects.

### 3.8. Additional Presumed Mechanisms of Action

In addition to the abovementioned activities, alternative anti-hypertensive mechanisms of action for citral and other lemongrass compounds are possible. Firstly, citral is known to act on several transient receptor potential (TRP) channels present in neurons, producing multiple responses on TRPV1, TRPV3, TRPV4, TRPM8, and TRPA1 channels. These receptors are also present in vascular cells, including endothelial and VSM cells [74,113,114]. Therefore, it is possible that citral can also act on these TRP channels to mediate vasorelaxation, which justifies the need for additional in vitro/ex vivo studies in order to clarify the possible role of these channels. Secondly, several bioactive compounds of lemongrass have been proposed to display diuretic activity, namely, saponins and flavonoids. Saponins are known to inhibit sodium/potassium-ATPase [115], and flavonoids also interfere with renal function [116]. Thirdly, several saponins are also known to inhibit components of the renin–angiotensin–aldosterone (RAA) axis in vitro [117], to which the blood-pressure-lowering effect might also be attributed. Fourthly, it is also possible that components from essential oils can saturate plasma membranes when inhaled or topically applied, which may affect the function of ion channels, transporters and enzymes [118]. Finally, considering the ever-increasing evidence of an inflammatory component in hypertension [119,120], it is also plausible that the anti-inflammatory activity of citral and lemongrass might also contribute to their anti-hypertensive potential.

## 4. Conclusions

Lemongrass extracts and essential oils, and the major compound citral, have all shown anti-hypertensive effects in animal models and in humans. Such effects are attributed to: (1) vasodilation through the potentiation of endothelial NO secretion and smooth muscle relaxation; (2) central nervous system-depressing activity, leading to a parasympathetic-mediated cardiac depression and to reduction in anxiety levels; and (3) potentiation of diuresis. Further studies are needed to identify other compounds in lemongrass with anti-hypertensive activity and explore additional pharmacological pathways. The safety profile of citral and lemongrass products, including pharmacological interactions, should also be better characterized in the future. This paper contributes to highlighting the anti-hypertensive potential of lemongrass, from which new drugs and/or plant-based medicines can be designed.

## Figures and Tables

**Table 1:** Composition of lemongrass essential oils from different geographical origins.

Authors	Geographical Origin	Part Used	Compounds (Percentage)
Goes et al. (2015) [46]	Brazil	Undisclosed	Geranial (41.84), neral (31.49), geranyl acetate (9.04), geraniol (6.00), 6-metil-5-hepten-2-one (1.73), (E)-caryophyllene (1.68), canfene (1.37), (E)-isocitral (1.34), γ-cadinene (1.13), linalool (1.00), (Z)-isocitral (0.56), δ-cadinene (0.43), limonene (0.27), (Z)-β-ocimene (0.26), α-pinene (0.24), borneol (0.19) and triciclene (0.18)
Zheljazkov et al. (2011) [47]	USA	Dried aerial parts	Geranial (25–53), neral (20–45), caryophyllene oxide (1.3–7.2), t-caryophyllene (0.3–2.2)
Chisowa et al. (1998) [48]	Zambia	Dried leaves	Geranial (39.0), neral (29.4), β-myrcene (18.0), geraniol (1.7), linalool (1.3), 1,8-cineole (1.0), 6-methyl-hept-5-en-2-one (0.8), undecan-2-one (0.5), (Z)-β-ocimene (0.4), citronellol (0.3), (E)-β-ocimene (0.3), α-terpineol (0.3), limonene (0.2), tridecan-2-one (0.2), α-pinene (trace), verbenol (trace)
Kasali et al. (2001) [49]	Nigeria	Fresh leaves	Geranial (33.7), neral (26.5), β-myrcene (25.3), neomenthol (3.3), linalyl acetate (2.3), (Z)-β-ocimene (1.0), nerol (0.8), (E)-β-ocimene (0.7), linalool (0.6), p-cymene (0.5), β-caryophyllene (0.3), citronellal (0.3), tetrahydrolinalool (0.3), fenchone (0.2), geraniol (0.2), myrcenol (0.2), β-patchoulene (0.2), camphor (0.1), 2,6-dimethyloctane (0.1), β-elemene (0.1), sabinol (0.1), trans-allo-ocimene (0.1)
Dutta et al. (2014) [50]	India	Fresh leaves	Neral (42.15), geranial (35.12), β-myrcene (12.39), citronellal (1.56), carveol (0.84), geraniol (0.75), limonene (0.38), caryophyllene (0.35), geranyl acetate (0.26), nerol (0.12)
Kamkaen et al. (2015) [51]	Thailand	Undisclosed	Geranial (44.6), neral (33.7), β-myrcene (5.2), selina-6-en-4-ol (1.4), Z-β-ocimene (0.7)

**Table 2:** Classes of polyphenol compounds identified in the lemongrass extracts.

Authors	Origin of Lemongrass	Type of Extract	Compounds
Asaolu et al. (2009) [67]	Nigeria	Water and ethanol extracts of powdered leaves	Alkaloids, saponins, tannins, anthraquinones, steroids, phenols and flavonoids
Ekpenyong et al. (2016) [69]	Nigeria	Water extract of powdered leaves	High levels of saponins, moderate levels of tannins, flavonoids and phenols and relatively low levels of anthraquinones, alkaloids and deoxy-sugars
Soares et. (2013) [70]	Angola	Water, methanol and ethanol extracts of powdered, shade-dried leaves	Aqueous—tannins, flavonoids and terpenoids Ethanolic—tannins, flavonoids and terpenoids Methanolic—tannins, flavonoids, alkaloids, steroids and terpenoids
Gazola et al. (2004) [71]	Brazil	Water extract of powdered dried leaves	Alkaloids, tannins and flavonoids
Campos et al. (2014) [72]	Chile	Water/methanol extract of air-dried leaves and stems	Chlorogenic acid, isoorientin, swertiajaponin, 6-C-pentosyl-8-C-hexosyl apigenin and luteolin C-rhamnosyl rhamnoside
Simões et al. (2020) [73]	Portugal	Water extract and fractions of dried leaves	Caffeic acid derivatives, p-coumaric acid derivatives, luteolin derivatives, apigenin derivatives and proanthocyanidins
Coelho et al. (2016) [65]	Portugal	Water extract of the whole dried plant	Phenolics (hydroxycinnamic acids—caffeic, p-coumaric, ferulic, chlorogenic and rosmarinic, flavonoid—quercitrin)
Somparn et al. (2018) [68]	Thailand	Water extract of the whole plant	Gallic acid, catechin, tannic acid, rutin, isoquercetin, hydroquinone, eriodictyol, quercetin

**Table 3:** Results of the vasorelaxant activity of citral in ex vivo and in vivo studies (WKYRs–Wistar-Kyoto rats; SHRs–spontaneously hypertensive rats).

Authors	Animal Species/Strain	Dose	Main Results
Devi et al. (2012) [87]	Thoracic aorta of SHRs (250–300 g; undisclosed age)	0.00624-6.24 mM	Attenuation of PE-, CaCl_2_- and KCl-mediated contraction in intact and endothelium-denuded vessels.
	Thoracic aorta of WKYRs (250–300 g; undisclosed age)		Failure to attenuate PE-precontracted vessels.
Pereira et al. (2013) [88]	Thoracic aorta of WKYRs (15–17 w.o., undisclosed weight)	10^−4^–6 mM	Attenuation of PE-, CaCl_2_- and KCl-mediated contraction.
Moreira (2013) [89]	Superior mesenteric artery of male Wistar rats (200–300 g, undisclosed age)	10^−5^–10^−2^ mM	Attenuation of PE-, CaCl_2_, KCl- and sodium orthovanadate-mediated contraction. Vasorelaxation inhibited by endothelium denudation.
	Male Wistar rats (200–300 g; undisclosed age) conscious or under thiopental anesthesia	1, 5, 10 and 20 mg/kg (i.v.)	Hypotension and bradycardia in conscious animals. L-NAME attenuated hypotension and atropine abolished bradycardia; hexamethonium and thiopental abolished both responses.

**Table 4:** Main results of ex vivo studies describing the cardiovascular activities of lemongrass products (m.o.—months old; SHRs—spontaneously hypertensive rats; WKYRs—Wistar-Kyoto rats; w.o.—weeks old).

Authors	Animal Species/Strain	Lemongrass Product (Concentration)	Main Results
Gazola et al. (2004) [71]	Isolated hearts from healthy male adult rats (undisclosed strain) (N = 7, ≈400 g)	Water extract of leaves (0.077, 0.77, 7.7 and 77 mg/mL)	Significant decrease in heart rate for all doses lasting 5 s; for the 38 mg dose only, the effect lasted for 15 s.
Runnie et al. (2004) [98]	Descending thoracic aorta from WKYRs (4 m.o.)	Methanol extract of powdered stalks (2.5–37.0 μg/mL for aorta assay; 100–10,000 μg/mL for mesenteric assay)	Vasorelaxation of NE-precontrated vessels, significantly decreased by incubation with NOLA but not by endothelium denudation.
	Superior mesenteric artery from WKYRs (4 m.o., undisclosed weight)		Vasorelaxation of PE-precontracted vessels, significantly decreased by incubation with NOLA and indomethacin.
Abeywardena et al. (2002) [99]	Descending thoracic aorta from WKYRs (4 m.o., undisclosed weight)	Methanol extract of powdered stalks (2.5–5 mg)	Vasorelaxation of PE-precontracted vessels, significantly decreased by incubation with NOLA or by endothelium denudation.
	Superior mesenteric artery from WKYRs (4 m.o., undisclosed weight)		Vasorelaxation of PE-precontracted vessels, unaffected by incubation with NOLA and indomethacin.
Devi et al. (2012) [87]	Thoracic aorta of WKYRs (250–300 g, undisclosed age)	Methanol extract of leaves, stems, and roots (1, 3, 10, 30, and 100 mg/mL)	Vasorelaxation of PE-precontracted vessels by all extracts.
	Thoracic aorta of SHRs (250–300 g, undisclosed age)		Vasorelaxation of PE-precontracted vessels by leaf and root extracts, the former being abolished by L-NAME or indomethacin. Root extract-mediated vasorelaxation was potentiated by indomethacin.
Martínez et al. (2020) [100]	Aorta of male adult Wistar rats (7–8 w.o.)	20% tincture of leaves (1, 3, 10, 30 and 100 mg/mL)	Vasorelaxation of endothelium-denuded PE- and KCl-precontracted vessels.
Campos et al. (2014) [72]	Human umbilical veins	Water/methanol extract of leaves and stems (10^−10^–10^−6^ M)	Inhibition of U46619-mediated vasoconstriction.
Simões et al. (2020) [73]	Human internal thoracic arteries	Infusion of leaves and fractions (0.002–0.2 mg/mL)	Infusion causes vasorelaxation, inhibited by indomethacin. Tannin fraction elicits the larger vasodilation.

## Data Availability

Not applicable.

## References

[1] Wierzejska E., Giernaś B., Lipiak A., Karasiewicz M., Cofta M., Staszewski R. (2020). A global perspective on the costs of hypertension: A systematic review. Arch. Med. Sci..

[2] Mancia G., De Backer G., Dominiczak A., Cifkova R., Fagard R., Germano G., Grassi G., Heagerty A.M., Kjeldsen S.E., Laurent S. (2007). 2007 Guidelines for the Management of Arterial Hypertension: The Task Force for the Management of Arterial Hypertension of the European Society of Hypertension (ESH) and of the European Society of Cardiology (ESC). J. Hypertens..

[3] Catalysing Ancient Wisdom and Modern Science for the Health of People and the Planet. https://www.who.int/initiatives/who-global-centre-for-traditional-medicine.

[4] Gopal N.M., Tejaswini J., Mantry S., Kumar S.A. (2014). International standards of medicinal plants. Int. J. Innov. Pharm. Sci. Res..

[5] Petrovska B.B. (2012). Historical review of medicinal plants’ usage. Pharmacogn. Rev..

[6] Bishop F.L., Yardley L., Lewith G.T. (2007). A systematic review of beliefs involved in the use of complementary and alternative medicine. J. Health Psychol..

[7] Barnes P.M., Bloom B., Nahin R.L. (2008). Complementary and alternative medicine use among adults and children: United States, 2007. Natl. Health Stat. Rep..

[8] Bertea C.M., Maffei M.E., Akhila A. (2010). The Genus Cymbopogon Botany, Including Anatomy, Physiology, Biochemistry, and Molecular Biology. Essential Oil Bearing Grasses The Genus Cymbopogon.

[9] Shah G., Shri R., Panchal V., Sharma N., Singh B., Mann A.S. (2011). Scientific basis for the therapeutic use of *Cymbopogon citratus*, stapf (Lemon grass). J. Adv. Pharm. Technol. Res..

[10] Machraoui M., Kthiri Z., Ben Jabeur M., Hamada W. (2018). Ethnobotanical and phytopharmacological notes on *Cymbopogon citratus* (DC.) Stapf. J. New Sci..

[11] Ravinder K., Pawan K., Gaurav S., Paramjot K., Gagan S., Appramdeep K. (2010). Pharmacognostical Investigation of *Cymbopogon citratus* (DC) Stapf. Sch. Res. Libr..

[12] Gaba J., Bhardwaj G., Sharma A. (2020). Lemongrass. Antioxidants in Vegetables and Nuts-Properties and Health Benefits.

[13] Ganjewala D. (2009). Cymbopogon essential oils: Chemical compositions and bioactivities. Int. J. Essent. Oil Ther..

[14] Vanisha S. (2016). Nambiar and Hema Matela Potential Functions of Lemon Grass (*Cymbopogon citratus*) in Health and Disease. Int. J. Pharm. Biol. Arch..

[15] Amirdivani S., Baba A.S. (2011). Changes in yogurt fermentation characteristics, and antioxidant potential and in vitro inhibition of angiotensin-1 converting enzyme upon the inclusion of peppermint, dill and basil. LWT Food Sci. Technol..

[16] Skaria B.P., Joy P.P., Mathew G., Mathew S., Joseph A. (2012). Lemongrass. Handb. Herbs Spices Second Ed..

[17] Ekpenyong C.E., Akpan E.E. (2017). Use of *Cymbopogon citratus* essential oil in food preservation: Recent advances and future perspectives. Crit. Rev. Food Sci. Nutr..

[18] Kamoga O.L.M., Kirabira J.B., Byaruhanga J.K. The Potential of Cymbopogon nardus in the Production of Pulp for Paper Industry. Proceedings of the International Conference on Computing, Mechanical and Electronics Engineering (ICCMEE’2015).

[19] Nur Firdaus M.Y., Osman H., Metselaar H.S.C., Rozyanty A.R. (2015). A simple method for the production of pure crystalline silica from lemon grass. BioResources.

[20] Bekele L.D., Zhang W., Liu Y., Duns G.J., Yu C., Jin L., Li X., Jia Q., Chen J. (2017). Preparation and Characterization of Lemongrass Fiber (Cymbopogon species) for Reinforcing Application in Thermoplastic Composites. Biosources.

[21] Babarinde A., Ogundipe K., Sangosanya K.T., Akintola B.D., Hassan A.O.E. (2016). Comparative study on the biosorption of Pb(II), Cd(II) and Zn(II) using Lemon grass (*Cymbopogon citratus*): Kinetics, isotherms and thermodynamics. Chem. Int..

[22] Alfa I.M., Dahunsi S.O., Iorhemen O.T., Okafor C.C., Ajayi S.A. (2014). Comparative evaluation of biogas production from Poultry droppings, Cow dung and Lemon grass. Bioresour. Technol..

[23] Wannissorn B., Jarikasem S., Soontorntanasart T. (1996). Antifungal activity of lemon grass oil and lemon grass oil cream. Phyther. Res..

[24] Pedroso R.B., Ueda-Nakamura T., Dias Filho B.P., Cortez D.A.G., Cortez L.E.R., Morgado-Díaz J.A., Nakamura C.V. (2006). Biological activities of essential oil obtained from *Cymbopogon citratus* on Crithidia deanei. Acta Protozool..

[25] Francisco V., Figueirinha A., Neves B.M., García-Rodríguez C., Lopes M.C., Cruz M.T., Batista M.T. (2011). *Cymbopogon citratus* as source of new and safe anti-inflammatory drugs: Bio-guided assay using lipopolysaccharide-stimulated macrophages. J. Ethnopharmacol..

[26] Khadri A., Serralheiro M.L.M., Nogueira J.M.F., Neffati M., Smiti S., Araújo M.E.M. (2008). Antioxidant and antiacetylcholinesterase activities of essential oils from Cymbopogon schoenanthus L. Spreng. Determination of chemical composition by GC-mass spectrometry and 13C NMR. Food Chem..

[27] Haque A.N.M.A., Remadevi R., Naebe M. (2018). Lemongrass (Cymbopogon): A review on its structure, properties, applications and recent developments. Cellulose.

[28] Darias V., Bravo L., Rabanal R., Mateo C.S., Luis R.M.G., Pérez A.M.H. (1989). New contribution to the ethnopharmacological study of the canary islands. J. Ethnopharmacol..

[29] Carbajal D., Casaco A., Arruzazabala L., Gonzalez R., Tolon Z. (1989). OF CYMBOPOGON or Cymbopogon Plant muterials Blood pressure measurement Measurement of diuretic activity Anti-inflammatory effect. J. Ethnopharmacol..

[30] Gómez Y.M., García C.J., Javier A., González D. (2010). Caña santa para el tratamiento de ancianos con hipertensión arterial / Lemongrass for treating aged persons with hypertension MsC. Medisan.

[31] Dzeufiet P.D.D., Mogueo A., Bilanda D.C., Aboubakar B.F.O., Tédong L., Dimo T., Kamtchouing P. (2014). Antihypertensive potential of the aqueous extract which combine leaf of Persea americana Mill. (Lauraceae), stems and leaf of *Cymbopogon citratus* (D.C) Stapf. (Poaceae), fruits of Citrus medical L. (Rutaceae) as well as honey in ethanol and sucrose experi. BMC Complement. Altern. Med..

[32] Locksley H., Fayez M., Radwan A., Chari V., Cordell G., Wagner H. (1982). Constituents of Local Plants. Planta Med..

[33] Leite J., Seabra M., Maluf E., Assolant K., Suchecki D., Tufik S., Klepacz S., Calil H., Carlini E. (1986). Pharmacology of lemongrass. III. Assessment of eventual toxic, hypnotic and anxiolytic effects on humans. J. Ethnopharmacol..

[34] Formigoni M.L.O.S., Lodder H.M., Filho O.G., Ferreira T.M.S., Carlini E.A. (1986). Pharmacology of lemongrass (*Cymbopogon citratus* Stapf). II. Effects of daily two month administration in male and female rats and in offspring exposed “in utero”. J. Ethnopharmacol..

[35] Ekpenyong C.E., Akpan E., Nyoh A. (2015). Ethnopharmacology, phytochemistry, and biological activities of *Cymbopogon citratus* (DC.) Stapf extracts. Chin. J. Nat. Med..

[36] Chrysant S.G., Chrysant G.S. (2017). Herbs Used for the Treatment of Hypertension and their Mechanism of Action. Curr. Hypertens. Rep..

[37] Law S., Lo C. (2021). “Lemongrass” and its applications for the treatment of hypertension. Infect. Dis. Herb. Med..

[38] Olorunnisola S.K., Asiyanbi H.T., Hammed A.M., Simsek S. (2014). Biological properties of lemongrass: An overview. Int. Food Res. J..

[39] Kamaruddin Z.H., Jumaidin R., Selamat M.Z., Ilyas R.A. (2021). Characteristics and Properties of Lemongrass (Cymbopogan Citratus): A Comprehensive Review. J. Nat. Fibers.

[40] Wifek M., Saeed A., Rehman R., Nisar S. (2016). Lemongrass: A review on its botany, properties, applications and active components Evaluation of the effects of Zinc on the chemical composition and biological activity of basil essential oil by using Raman spectroscopy View project Lemongrass: A review. Int. J. Chem. Biochem. Sci..

[41] Karami S., Yargholi A., Lamardi S.N.S., Soleymani S., Shirbeigi L., Rahimi R. (2021). A review of ethnopharmacology, phytochemistry and pharmacology of cymbopogon species. Res. J. Pharmacogn..

[42] Manvitha B.B.K. (2014). Review on pharmacological activity of *Cymbopogon citratus*. Int. J. Herb. Med..

[43] Carlson L.H.C., Machado R.A.F., Spricigo C.B., Pereira L.K., Bolzan A. (2001). Extraction of lemongrass essential oil with dense carbon dioxide. J. Supercrit. Fluids.

[44] Majewska E., Kozlowska M., Gruczynska-Sekowska E., Kowalska D., Tarnowska K. (2019). Lemongrass (*Cymbopogon citratus*) essential oil: Extraction, composition, bioactivity and uses for food preservation—A review. Pol. J. Food Nutr. Sci..

[45] Aftab K., Ali M.D., Aijaz P., Beena N., Gulzar H.J., Sheikh K., Sofia Q., Abbas S.T. (2011). Determination of different trace and essential element in lemon grass samples by X-ray fluorescence spectroscopy technique. Int. Food Res. J..

[46] Goes T.C., Ursulino F.R.C., Almeida-Souza T.H., Alves P.B., Teixeira-Silva F. (2015). Effect of lemongrass aroma on experimental anxiety in humans. J. Altern. Complement. Med..

[47] Zheljazkov V.D., Cantrell C.L., Astatkie T., Cannon J.B. (2011). Lemongrass productivity, oil content, and composition as a function of nitrogen, sulfur, and harvest time. Agron. J..

[48] Chisowa E.H., Hall D.R., Farman D.I. (1998). Volatile constituents of the essential oil of *Cymbopogon citratus* Stapf grown in Zambia. Flavour Fragr. J..

[49] Kasali A.A., Oyedeji A.O., Ashilokun A.O. (2001). Volatile leaf oil constituents of *Cymbopogon citratus* (DC) Stapf. Flavour Fragr. J..

[50] Dutta D., Kumar P., Nath A., Verma N., Gangwar B. (2014). Qualities of lemongrass (*Cymbopogan citratus*) essential oil at different drying conditions. Int. J. Agric. Environ. Biotechnol..

[51] Kamkaen N., Ruangrungsi N., Patalung N.N., Watthanachaiyingcharoen R. (2015). Physiological and Psychological Effects of Lemongrass and Sweet Almond Massage Oil. J. Health Res..

[52] Barbosa L.C.A., Pereira U.A., Martinazzo A.P., Maltha C.R.Á., Teixeira R.R., Melo E.D.C. (2008). Evaluation of the chemical composition of Brazilian commercial *Cymbopogon citratus* (D.C.) stapf samples. Molecules.

[53] Ekpenyong C.E., Akpan E.E., Daniel N.E. (2014). Phytochemical Constituents, Therapeutic Applications and Toxicological Profile of *Cymbopogon citratus* Stapf (DC) Leaf Extract. J. Pharmacogn. Phytochem..

[54] Mansour, Fikry R.M., Saad M.M., Mohamed A.M. (2014). Chemical composition, antioxidant and antimicrobial activity of (*Cymbopogon citratus*) essential oil cultivated in Madinah Monawara, Saudi Arabia and its comparison to the Egyptian chemotype. Int. J. Food Nutr. Sci..

[55] Farias P.K.S., Lopes Silva J.C.R., de Souza C.N., da Fonseca F.S.A., Brandi I.V., Martins E.R., Azevedo A.M., de Almeida A.C. (2019). Antioxidant activity of essential oils from condiment plants and their effect on lactic cultures and pathogenic bacteria. Cienc. Rural.

[56] Moutassem D., Belabid L., Bellik Y., Ziouche S., Baali F. (2019). Efficacy of essential oils of various aromatic plants in the biocontrol of Fusarium wilt and inducing systemic resistance in chickpea seedlings. Plant Prot. Sci..

[57] Matasyoh J.C., Wagara I.N., Nakavuma J.L., Kiburai A.M. (2011). Chemical composition of *Cymbopogon citratus* essential oil and its effect on mycotoxigenic Aspergillus species. Afr. J. Food Sci..

[58] Mbili N.C., Opara U.L., Lennox C.L., Vries F.A. (2017). Citrus and lemongrass essential oils inhibit Botrytis cinerea on “golden delicious”, “pink lady” and “granny Smith” apples. J. Plant Dis. Prot..

[59] Abegaz B., Yohannes P.G., Dieter R.K. (1983). Constituents of the essential oil of ethiopian *Cymbopogon citratus* staff. J. Nat. Prod..

[60] Crawford M., Hanson S.W., Koker M.E.S. (1975). The structure of cymbopogone, a novel triterpenoid from lemongrass. Tetrahedron Lett..

[61] Hanson S.W., Crawford M., Koker M.E.S., Menezes F.A. (1976). Cymbopogonol, a new triterpenoid from *Cymbopogon citratus*. Phytochemistry.

[62] Shaikh M.N., Suryawanshi Y.C., Mokat D.N. (2019). Volatile Profiling and Essential Oil Yield of *Cymbopogon citratus* (DC.) Stapf Treated with Rhizosphere Fungi and Some Important Fertilizers. J. Essent. Oil-Bearing Plants.

[63] Verma R.K., Verma R.S., Chauhan A., Bisht A. (2015). Evaluation of essential oil yield and chemical composition of eight lemongrass (Cymbopogon spp.) cultivars under Himalayan region. J. Essent. Oil Res..

[64] Tajidin N., Ahmad S., Rosenani A., Azimah H., Munirah M. (2012). Chemical composition and citral content in lemongrass (*Cymbopogon citratus*) essential oil at three maturity stages. Afr. J. Biotechnol..

[65] Coelho M., Rocha C., Cunha L.M., Cardoso L., Alves L., Lima R.C., Pereira M.J., Campos F.M., Pintado M. (2016). Influence of harvesting factors on sensory attributes and phenolic and aroma compounds composition of *Cymbopogon citratus* leaves infusions. Food Res. Int..

[66] Hanaa A.R.M., Sallam Y.I., El-Leithy A.S., Aly S.E. (2012). Lemongrass (*Cymbopogon citratus*) essential oil as affected by drying methods. Ann. Agric. Sci..

[67] Asaolu M., Oyeyemi O., Olanlokun J. (2009). Chemical Compositions, Phytochemical Constituents and in vitro Biological Activity of Various Extracts of *Cymbopogon citratus*. Pak. J. Nutr..

[68] Somparn N., Saenthaweeuk S., Naowaboot J., Thaeomor A., Kukongviriyapan V. (2018). Effect of lemongrass water extract supplementation on atherogenic index and antioxidant status in rats. Acta Pharm..

[69] Ekpenyong C., Osim E., America S. (2016). Changes in blood pressure indices in normotensive adults after the consumption of lemongrass tea. J. Coast. Life Med..

[70] Soares M.O., Alves R.C., Pires P.C., Oliveira M.B.P.P., Vinha A.F. (2013). Angolan *Cymbopogon citratus* used for therapeutic benefits: Nutritional composition and influence of solvents in phytochemicals content and antioxidant activity of leaf extracts. Food Chem. Toxicol..

[71] Gazola R., MacHado D., Ruggiero C., Singi G., Alexandre M.M. (2004). Lippia alba, Melissa officinalis and *Cymbopogon citratus*: Effects of the aqueous extracts on the isolated hearts of rats. Pharmacol. Res..

[72] Campos J., Schmeda-Hirschmann G., Leiva E., Guzmán L., Orrego R., Fernández P., González M., Radojkovic C., Zuñiga F.A., Lamperti L. (2014). Lemon grass (*Cymbopogon citratus* (D.C) Stapf) polyphenols protect human umbilical vein endothelial cell (HUVECs) from oxidative damage induced by high glucose, hydrogen peroxide and oxidised low-density lipoprotein. Food Chem..

[73] Simões D.M., Malheiros J., Antunes P.E., Figueirinha A., Cotrim M.D., Fonseca D.A. (2020). Vascular activity of infusion and fractions of *Cymbopogon citratus* (DC) Stapf. in human arteries. J. Ethnopharmacol..

[74] Stotz S.C., Vriens J., Martyn D., Clardy J., Clapham D.E. (2008). Citral sensing by TRANSient receptor potential channels in dorsal root ganglion neurons. PLoS ONE.

[75] Diliberto J.J., Usha G., Birnbaum L.S. (1988). Disposition of citral in male Fischer rats. Drug Metab. Dispos..

[76] Baldwin J.R., Witiak D.T., Feller D.R. (1977). Studies on the biotransformation and excretion of 14C-clofibrate in the rat. Fed. Proc..

[77] Diliberto J.J., Srinivas P., Overstreet D., Usha G., Burka L.T., Birnbaum L.S. (1990). Metabolism of citral, an α,β-unsaturated aldehyde, in male F344 rats. Drug Metab. Dispos..

[78] Gaworski C., Vollmuth T., York R., Heck J., Arany C. (1992). Developmental toxicity evaluation of inhaled citral in Sprague-Dawley rats. Food Chem. Toxicol..

[79] Nakamura Y., Miyamoto M., Murakami A., Ohigashi H., Osawa T., Uchida K. (2003). A phase II detoxification enzyme inducer from lemongrass: Identification of citral and involvement of electrophilic reaction in the enzyme induction. Biochem. Biophys. Res. Commun..

[80] Li C.C., Yu H.F., Chang C.H., Liu Y.T., Yao H.T. (2018). Effects of lemongrass oil and citral on hepatic drug-metabolizing enzymes, oxidative stress, and acetaminophen toxicity in rats. J. Food Drug Anal..

[81] Song Y., Zhao H., Liu J., Fang C., Miao R. (2016). Effects of Citral on Lipopolysaccharide-Induced Inflammation in Human Umbilical Vein Endothelial Cells. Inflammation.

[82] Bachiega T.F., Sforcin J.M. (2011). Lemongrass and citral effect on cytokines production by murine macrophages. J. Ethnopharmacol..

[83] Katsukawa M., Nakata R., Takizawa Y., Hori K., Takahashi S., Inoue H. (2010). Citral, a component of lemongrass oil, activates PPAR α and γ and suppresses COX-2 expression. Biochim. Biophys. Acta Mol. Cell Biol. Lipids.

[84] Safaeian L., Sajjadi S.E., Montazeri H., Ohadi F., Javanmard S. (2020). Citral protects human endothelial cells against hydrogen peroxide-induced oxidative stress. Turk. J. Pharm. Sci..

[85] Sforcin J.M., Amaral J.T., Fernandes A., Sousa J.P.B., Bastos J.K. (2009). Lemongrass effects on IL-1β and IL-6 production by macrophages. Nat. Prod. Res..

[86] Mediesse F.K., Boudjeko T., Hasitha A., Gangadhar M., Mbacham W.F., Yogeeswari P. (2018). Inhibition of lipopolysaccharide (LPS)-induced neuroinflammatory response by polysaccharide fractions of Khaya grandifoliola (C.D.C.) stem bark, Cryptolepis sanguinolenta (Lindl.) Schltr and *Cymbopogon citratus* Stapf leaves in raw 264.7 macrophages and U8. BMC Complement. Altern. Med..

[87] Devi R.C., Sim S.M., Ismail R. (2012). Effect of *Cymbopogon citratus* and citral on vascular smooth muscle of the isolated thoracic rat aorta. Evidence-Based Complement. Altern. Med..

[88] Pereira S.L., Marques A.M., Sudo R.T., Kaplan M.A.C., Zapata-Sudo G. (2013). Vasodilator activity of the essential oil from aerial parts of Pectis brevipedunculata and its main constituent citral in rat aorta. Molecules.

[89] Moreira F. (2013). Cardiovascular Effects of the Citral, Major Monoterpene of the Essential oil of *Cymbopogon citratus*, in Rats. Ph.D. Thesis.

[90] Wistar Kyoto (WKY) Rat|Charles River. https://www.criver.com/products-services/find-model/wistar-kyoto-wky-rat?region=3616.

[91] Höferl M., Krist S., Buchbauer G. (2006). Chirality influences the effects of linalool on physiological parameters of stress. Planta Med..

[92] Yayama K., Sasahara T., Ohba H., Funasaka A., Okamoto H. (2014). Orthovanadate-induced vasocontraction is mediated by the activation of Rho-kinase through Src-dependent transactivation of epidermal growth factor receptor. Pharmacol. Res. Perspect..

[93] Ribeiro-Filho H.V., De Souza Silva C.M., De Siqueira R.J., Lahlou S., Dos Santos A.A., Magalhães P.J.C. (2016). Biphasic cardiovascular and respiratory effects induced by β-citronellol. Eur. J. Pharmacol..

[94] Bastos J.F.A., Moreira Í.J.A., Ribeiro T.P., Medeiros I.A., Antoniolli A.R., De Sousa D.P., Santos M.R.V. (2010). Hypotensive and vasorelaxant effects of citronellol, a monoterpene alcohol, in rats. Basic Clin. Pharmacol. Toxicol..

[95] Andrade F.C., Mota M.M., Barreto A.S., Sousa D.P., Quintans-Junior L.J., Santos M.R.V. (2012). Antihypertensive therapeutic potential of citronellal. Lat. Am. J. Pharm..

[96] Kundu S., Shabir H., Basir S.F., Khan L.A. (2014). Inhibition of As(III) and Hg(II) caused aortic hypercontraction by eugenol, linalool and carvone. J. Smooth Muscle Res..

[97] Kang P., Seol G.H. (2015). Linalool elicits vasorelaxation of mouse aortae through activation of guanylyl cyclase and K+ channels. J. Pharm. Pharmacol..

[98] Runnie I., Salleh M.N., Mohamed S., Head R.J., Abeywardena M.Y. (2004). Vasorelaxation induced by common edible tropical plant extracts in isolated rat aorta and mesenteric vascular bed. J. Ethnopharmacol..

[99] Abeywardena M., Runnie I., Nizar M., Suhaila M., Head R. (2002). Polyphenol-enriched extract of oil palm fronds (Elaeis guineensis) promotes vascular relaxation via endothelium-dependent mechanisms. Asia Pac. J. Clin. Nutr..

[100] Galán Martínez L. (2020). Effect of a tincture of *Cymbopogon citratus* leaves on vascular smooth muscle of rats. Int. J. Pharma Sci. Res..

[101] Jutabha P., Chomdej B. (1995). Effect of *Cymbopogon citratus* Stapf. on renal functions in dogs. Chula Med. J..

[102] Singi G., Damasceno D.D., Silva G.A. (2005). Artigo Efeitos agudos dos extratos hidroalcoólicos do alho. Rev. Bras. Farmacogn..

[103] Tcheutchoua Y.C., Bilanda D.C., Dzeufiet P.D.D., Djunie Neali O.C., Owona P.E., Bidingha R.À.G., Ngapout R.F., Mbolang L.N., Noubom M., Dimo T. (2022). Preventive Potential of the Aqueous Extract of the Mixture of Bidens pilosa (Asteraceae) and *Cymbopogon citratus* (Poaceae) Aerial Parts on Hypertension Induced by a Chronic Salt and Alcohol Consumption on the Rats. Evidence-Based Complement. Altern. Med..

[104] Moreira F.V., Bastos J.F.A., Blank A.F., Alves P.B., Santos M.R.V. (2010). Chemical composition and cardiovascular effects induced by the essential oil of *Cymbopogon citratus* DC. Stapf, Poaceae, in rats. Rev. Bras. Farmacogn..

[105] Arome D., Enegide C., Ameh S. (2014). Pharmacological evaluation of anxiolytic property of aqueous root extract of *Cymbopogon citratus* in mice. Chron. Young Sci..

[106] Ekpenyong C.E. (2018). Lemongrass tea consumption and changes in Acid-Base Balance and Electrolyte homeostasis. Arch. Food Nutr. Sci..

[107] Sobha R. (2014). The Effect of Lemongrass Decoction in Reduction of Blood Pressure Among Individuals With Hypertension in Selected Community Area, Kerala. Ph.D. Thesis.

[108] Patin R., Kanlayavattanakul M., Lourith N. (2010). Aromatherapy and Essential Oils in Thai Spa Business. Isan J. Pharm. Sci..

[109] Sriraksa N., Kaewwongse M., Phachonpai W., Hawiset T. (2018). Effects of Lemongrass (*Cymbopogon citratus*) Essential Oil Inhalation on Cognitive Performance and Mood in Healthy Women. Thai Pharm. Health Sci. J..

[110] Shimono K., Oka H., Suzuki M., Senda K., Komai S. (2010). Aromatic Antihypertensive Agent, and Method for Lowering Blood Pressure in Mammals. U.S. Patent.

[111] Siahaan R., Rahardjo T.B., Ranti A. (2014). Effectiveness of Indonesian Essential Oil Mixture of Lemongrass, Cananga, and Patchouli in Relaxation through Inhalation: A Clinical Test on Healthy Woman with High Potential for Stress. Makara J. Health Res..

[112] Blaak J., Staib P. (2022). An updated review on efficacy and benefits of sweet almond, evening primrose and jojoba oils in skin care applications. Int. J. Cosmet. Sci..

[113] Filosa J.A., Yao X., Rath G. (2013). TRPV4 and the regulation of vascular tone. J. Cardiovasc. Pharmacol..

[114] Vincent F., Acevedo A., Nguyen M.T., Dourado M., DeFalco J., Gustafson A., Spiro P., Emerling D.E., Kelly M.G., Duncton M.A.J. (2009). Identification and characterization of novel TRPV4 modulators. Biochem. Biophys. Res. Commun..

[115] Rhiouani H., Settaf A., Lyoussi B., Cherrah Y., Lacaille-Dubois M.A., Hassar M. (1999). Effects of saponins from Herniaria glabra on blood pressure and renal function in spontaneously hypertensive rats. Therapie.

[116] Jouad H., Haloui M., Rhiouani H., Hilaly J.E., Eddouks M. (2001). Ethnobotanical survey of medicinal plants used for the treatment of diabetes, cardiac and renal diseases in the North centre region of Morocco (Fez-Boulemane). J. Ethnopharmacol..

[117] Chen M., Long Z., Wang Y., Liu J., Pian H., Wang L., Chen Z. (2013). Protective effects of saponin on a hypertension target organ in spontaneously hypertensive rats. Exp. Ther. Med..

[118] Buchbauer G., Jirovetz L. (1994). Aromatherapy—use of fragrances and essential oils as medicaments. Flavour Fragr. J..

[119] Xiao L., Harrison D.G. (2020). Inflammation in Hypertension. Can. J. Cardiol..

[120] Dixon D.L., Wohlford G.F., Abbate A. (2020). Inflammation and Hypertension Causal or Not?. Can. J. Cardiol..

